# Possible Predictive Factors for In-hospital Cardiac Arrest in Patients with Cancer: A Retrospective Single Center Study

**DOI:** 10.7759/cureus.2828

**Published:** 2018-06-18

**Authors:** Muhammad Sardar, Nasreen Shaikh, Saad Ullah Malik, Faiz Anwer, Patrick Lee, David Sharon, Margaret HH Eng

**Affiliations:** 1 Internal Medicine, Monmouth Medical Center, Long Branch, USA; 2 Department of Internal Medicine, Monmouth Medical Center, Long Branch, USA; 3 Hematology Oncology, University of Arizona, Tuscon, USA; 4 Hematology and Oncology, University of Arizona, Tucson, USA; 5 Hematology Oncology, Monmouth Medical Center, Long Branch, USA

**Keywords:** cardiac arrest, predictors, cancer

## Abstract

Background: Despite cancer being the second most common cause of death in the United States, more people are living longer after the diagnosis of cancer than before. Healthcare workers will be treating an increasing number of patients with cancer. Various studies have identified predictors of cardiac arrest in the general population, however, none have been done to identify such factors in cancer patients who form a more vulnerable group with lower survival rate following cardiac arrest.

Methods: We retrospectively analysed charts of all patients with active cancer who experienced in-hospital cardiac arrest (IHCA) and underwent cardio-pulmonary resuscitation (CPR) from January 2015 to December 2017 at our hospital (n=44, group A). We compared this group to 44 consecutive patients with active cancer admitted to the oncology unit who did not experience cardiac arrest (n=44, group B). We excluded patients in remission.

Results: Both the groups were comparable in terms of age (69 ± 14 vs 68 ± 15, p=0.776) and gender distribution (50% vs 56% males, p=0.521). Prevalence of coronary artery disease (CAD) (25% vs 11%, p=0.097), hypertension (68% vs 66%, p=0.821), hyperlipidaemia (34% in both groups, p=1.000), tobacco abuse (18% vs 27%, p=0.308), and diabetes mellitus (34% vs 23%, p=0.237) was not significantly different between the two groups. Group with cardiac arrest had significantly higher alanine aminotransferase (100 U/L ± 150 vs 47 U/L ± 87, p=0.043), alkaline phosphatase (288 U/L ± 512 vs 118 U/L ± 80, p=0.032), creatinine (1.8 mg/dl ± 1.74 vs 1.1 mg/dl ± 0.76, p=0.023), international normalised ratio (INR) (2.1 ± 1.5 vs 1.2 ± 0.5, p=0.005), and lower estimated -glomerular filtration rate (43 mL/min/1.73m^2^ ± 17 vs 51 mL/min/1.73m^2^ ± 15, p=0.022) on admission. Group A also had significantly higher incidence of sepsis during the hospital course as compared to group B (30% vs 2%, p<0.001). In group A, 11.4% survived to discharge as compared to 95.5% in group B. Significantly higher number of patients in group B were taking chemotherapy (77.27% vs 34.09%, p=0.000046) and radiation therapy (65.9% vs 22.72%, p=0.000046) as compared to group A.

Conclusion: Cancer patients who experienced IHCA had worse renal and hepatic function; they were frequently diagnosed with sepsis and had similar cardiovascular risk factors as compared to cancer patients who did not experience cardiac arrest. Furthermore, a higher number of patients with active cancer who did not experience cardiac arrest were on chemotherapy, immunotherapy or radiation therapy.

## Introduction

According to the Centers for Disease Control and Prevention (CDC), cancer was the second leading cause of death in 2015 with 595,930 deaths preceded only by heart disease as the number one cause of death [1]. In spite of cancer being the second leading cause of death, people diagnosed with cancer in the United States are living longer than before [2]. Healthcare providers will be expected to treat an increasing number of cancer patients in their practice; hence they should be able to identify patients with high risk of mortality.

Several scoring systems have been developed to identify patients on regular medical floors at high risk for adverse events. National Early Warning Score (NEWS), developed in 2012 by the Royal College of Physicians, is one such risk stratification system that takes into account vital signs (heart rate, respiratory rate, temperature, systolic blood pressure, oxygen saturation, and oxygen supplementation) and mental status to identify patients at risk of deterioration [3]. Electronic-Cardiac Arrest Risk Triage (e-CART) is a scoring system that incorporates ward vital signs as well as laboratory results and demographics for combined prediction of cardiac arrest, intensive care unit (ICU) transfer, and death [4]. Multiple studies have validated their use in real-time [5]. These scoring systems predict the probability of an adverse event in the general patient population and help physicians make timely interventions to prevent cardiac arrest or death.

Patients with cancer are a more vulnerable group compared to the general patient population. Survival rates after in-hospital cardiac arrest (IHCA) in patients with advanced cancer are lower than the rest of the population. In a study by Bruckle et al., 7.4% patients with advanced cancer who had IHCA survived to discharge as compared to 13.4% without advanced cancer [6]. A knowledge of predictors of cardiac arrest in this population would help physicians act in a timely manner to avert this adverse event.

No study has been done to identify factors predicting cardiac arrest in cancer patients. We aim to compare demographics, vital signs, laboratory results, electrocardiogram (EKG) characteristics, and cancer characteristics of patients with active cancer who suffered IHCA to patients with active cancer who did not experience IHCA at our hospital.

## Materials and methods

Patients who experienced cardiac arrest and underwent cardio-pulmonary resuscitation (CPR) at Monmouth Medical Center from January 2015 to December 2017 were identified. After thorough chart analysis, those patients who had active cancer were included in the study. Active cancer was defined as patients having a documented objective evidence of cancer and who were not in remission. We excluded patients on hospice care, patients who experienced cardiac arrest in the field, and those with the order of do not resuscitate. These patients formed group A (n=44). Group B consisted of 44 consecutive patients with active cancer admitted to the oncology unit who did not experience cardiac arrest from November 2017 to December 2017.

Baseline demographic and clinical characteristics, type and stage of cancer, medications, vital signs, complete blood count and comprehensive metabolic panel on the day of admission, and presence or absence of sepsis during hospital course were compared between the two groups. 12-lead EKG on admission was also compared. Surgical resection of the primary tumor, treatment with chemotherapy or radiation therapy was considered positive if it was within six months of admission.

Categorical variables were expressed as numbers with percentages and continuous variables were expressed as mean ± standard deviation. Continuous variables were compared between the two groups using Student's t-test or Mann-Whitney U test. Categorical variables were compared using chi-squared test or Fischer's exact test. Statistical significance was defined as a p value <0.05. All data were analysed using SPSS 20.0 (SPSS Inc Chicago, Illinois, USA).

## Results

Among 209 patients with IHCA followed by cardiopulmonary resuscitation from January 2015 to December 2017, 44 patients had active cancer (21.05%). The mean age of group with cardiac arrest (group A) was 69.14±13.91 compared to 68.25±15.24 in the group with the absence of cardiac arrest (group B), the difference was not significant. There was no significant difference in the prevalence of coronary artery disease (CAD), hyperlipidaemia, hypertension, diabetes mellitus, tobacco abuse, chronic obstructive pulmonary disease (COPD), and congestive heart failure (CHF) between the two groups. Significantly higher number of patients developed sepsis during hospital course in the group with cardiac arrest (29.54% vs 2.27%, p=0.00047). Table 1 summarizes the baseline demographic and clinical characteristics.

**Table 1:** Patient characteristics COPD: chronic obstructive pulmonary disease; CHF: congestive heart failure.

Variable	Cardiac arrest (n=44)	No cardiac arrest (n=44)	P value
Age	69.14 ± 13.91	68.25 ± 15.24	0.776
Male sex	22 (50%)	25 (56.8%)	0.521
Coronary artery disease	11 (25%)	5 (11.4%)	0.097
Hyperlipidemia	15 (34.1%)	15 (34.1%)	1.000
Hypertension	30 (68.2%)	29 (65.9%)	0.821
Diabetes mellitus	15 (34.1%)	10 (22.7%)	0.237
COPD	4 (9.1%)	5 (11.5%)	0.725
Peripheral vascular disease	5 (11.4%)	1 (2.3%)	0.091
Pulmonary embolism	5 (11.4%)	1 (2.3%)	0.091
CHF	8 (18.2%)	4 (9.1%)	0.214
Sepsis	13 (29.5%)	1 (2.3%)	0.0004
Tobacco abuse	8 (18.2%)	1 (2.3%)	0.308

Cancer characteristics and treatment

Both groups had 25% (11/44) patients with haematological malignancy and 75% (33/44) patients with solid tumours. Metastatic disease was seen in 29.5% of patients who had cardiac arrest compared to 47.8% of the patients who did not have cardiac arrest, however, results were not statistically significant. 77.3% of patients who did not have cardiac arrest were on active treatment (chemotherapy and/or immunotherapy) in the six months preceding admission compared to 34.1% who experienced cardiac arrest, the difference was statistically significant, p=0.000046. Similarly, 15.9% in group A had radiation therapy in the six months preceding admission as compared to 65.9% in group B, the difference was significant, p=0. 00002. Results are summarized in Table 2.

**Table 2:** Cancer characteristics and treatment

Variables	Cardiac arrest (n=44)	No cardiac arrest (n=44)	P Value
Hematological malignancy	11 (25%)	11 (25%)	1.000
Solid malignancy	33 (75%)	33 (75%)	1.000
Metastasis	13 (29.5%)	21 (47.8%)	0.067
Chemotherapy ± immunotherapy	15 (34.1%)	34 (77.3%)	0.000046
Radiotherapy	7 (15.9%)	29 (65.9%)	0.000002
Surgery	8 (18.2%)	9 (20.5%)	0.787

Vital signs and EKG on admission

Blood pressure, heart rate, and temperature were not significantly different between the two groups. The group with cardiac arrest had significantly higher respiratory rates (24.5 ± 15.4 vs 19.4 ± 3.1, p=0.034) and lower oxygen saturation (95.4 ± 3.6 vs 97 ± 2.5, p=0.014) however oxygen supplementation was similar in both groups. Patients who did not have cardiac arrest were more often in normal sinus rhythm on admission based on 12-lead EKG (1.8% in group A vs 50% in group B, p value=0.000085). The results are summarized in Table 3 and Table 4.

**Table 3:** Vital signs

Variable	Cardiac arrest (n=44)	No cardiac arrest (n=44)	P Value
Systolic blood pressure (mmHg)	116.6 ± 26.2	125.5 ± 26	0.116
Diastolic blood pressure (mmHg)	67.1 ± 18.2	68.8 ± 15.2	0.631
Heart rate	97.1 ± 29.2	92.5 ± 21.7	0.411
Respiratory rate	24.5 ± 15.4	19.4 ± 3.1	0.034
Oxygen saturation	95.4 ± 3.6	97 ± 2.5	0.014
Temperature	97.9 ± 1.4	98.3 ± 1.5	0.189

**Table 4:** Electrocardiogram (EKG) characteristics n is higher than 44 in each group as some patients may have more than one finding on their EKG.

Variable	Cardiac arrest (n=44)	No cardiac arrest (n=44)	P value
Normal Sinus rhythm	5 (1.8%)	22 (50%)	0.000085
Sinus tachycardia	15 (34.1%)	13 (29.5%)	0.209
Sinus bradycardia	2 (4.5%)	1 (2.3%)	0.557
Heart block (1^st^ /2^nd^ /3^rd^ degree)	2 (4.5%)	0	1.000
Bundle branch block (right or left)	7 (15.9%)	2 (4.5%)	1.791
Atrial arrhythmia	10 (22.7%)	3 (6.8%)	1.222
Ventricular arrhythmia	4 (9.1%)	6 (13.6%)	0.715
Non-specific ST/T wave changes	6 (13.6%)	2 (4.5%)	1.380
Paced rhythm	1 (2.3%)	2 (4.5%)	0.557

Laboratory tests

There was no significant difference in white blood cell count, platelet count and hemoglobin between the two groups on admission. International normalised ratio (INR) was significantly higher in the group with cardiac arrest (2.1 ± 1.6 vs 1.3 ± 0.5, p=0.005). Sodium, potassium and bicarbonate levels were not significantly different between the two groups. In the group with cardiac arrest, magnesium (2.2 vs 1.4, p=0.002) and phosphorous (4.1 vs 3.1, p=0.041) were significantly higher. Alanine aminotransferase (100.4 ± 149.9 vs 46.8 ± 86.8, p=0.043) and alkaline phosphatase (288 ± 512.8 vs 118.3 ± 80.2, 0.032) were significantly higher in group A. Aspartate aminotransferase was not significantly different. Patients with cardiac arrest also had significantly higher serum creatinine on admission (1.8 ± 1.7 vs 1.2 ± 0.8 p=0.023), lower estimated glomerular filtration rate (44 ± 16.8 vs 51.8 ± 14.6, p=0.022) and higher blood urea nitrogen (42.6 ± 29.8 vs 26.3 ± 17.5, p=0.002). Results are summarized in Table 5.

**Table 5:** Laboratory data WBC: white blood cell count; INR: international normalised ratio; AST: aspartate transaminase; ALT: alanine transaminase; eGFR: estimated glomerular filtration rate; BUN: blood urea nitrogen; BNP: brain natriuretic peptide.

Variable	Cardiac arrest (n=44)	No cardiac arrest (n=44)	P Value
WBC	29.6 ± 106.2	14.0 ± 26	0.351
Hemoglobin	10.9 ± 2.6	10.3 ± 2.7	0.800
Platelets	186 ± 127.0	225.9 ± 138.9	0.162
Sodium	137.7 ± 5.1	137.6136 ± 6.2	0.926
Potassium	4.4 ± 0.8	4.4±0.6	0.988
Bicarbonate	26.3 ±7.5	27.4 ± 4.6	0.378
Magnesium	2.2 ± 0.49	1.4 ± 0.4	0.002
Phosphorus	4.1 ± 2.2	3.1 ± 0.9	0.041
INR	2.1 ± 1.6	1.3 ± 0.5	0.005
AST	177.4 ± 375.3	75.5 ± 169.0	0.104
ALT	100.4 ± 149.9	46.8 ± 86.8	0.043
Alkaline phosphatase	288 ± 512.8	118.3 ± 80.2	0.032
Creatinine	1.8 ± 1.7	1.2 ± 0.8	0.023
eGFR	44 ± 16.8	51.8 ± 14.6	0.022
BUN	42.6 ± 29.8	26.3 ± 17.5	0.002
Troponins	0.73 ± 1.9	0.14 ± 0.48	0.238
BNP	821.6 ± 594.0	88 ± 46.2	0.060

Medications

There was no significant difference in the medications that the patients were taking on admission namely antiplatelet, anticoagulants, beta blockers, calcium channel blockers (dihydropyridines & nondihydropyridines), angiotensin-converting enzyme inhibitors/angiotensin receptor blockers, and statins. Table 6 shows the medications in both groups.

**Table 6:** Medications ACE-I/ARBs: angiotensin converting enzyme inhibitor/angiotensin receptor blockers.

Variable	Cardiac arrest (n=44)	No cardiac arrest (n=44)	P Value
Antiplatelets	13 (29.54%)	9 (20.45%)	0.325
Anticoagulants	10 (22.27%)	8 (18.18%)	0.597
Beta blockers	23 (52.27%)	15 (34.09%)	0.085
Calcium channel blockers	8 (18.18%)	11 (25%)	0.437
Statins	13(29.54%)	12 (27.27%)	0.813
ACE-I/ARBs	13 (29.54%)	11 (25%)	0.632

Characteristics of cardiac arrest

50% of the cardiac arrest rhythms were pulseless electrical activity, 31.81% were asystole and 15.9% were ventricular fibrillation or pulseless ventricular tachycardia. 11.36% of the patients with cardiac arrest survived to discharge. However, one patient died within three months after discharge. In comparison, 95.5% patients in the group B survived to discharge.

## Discussion

Our study aimed to identify factors that could predict the occurrence of IHCA in patients with active cancer so that pre-emptive interventions can be made to avert this adverse event. We found that evaluation of vital signs, laboratory results, and EKG findings can help identify patients at risk of developing IHCA.

Cancer patients who experienced IHCA were found to have worse hepatic and renal function on admission as compared to those who did not have IHCA. Various studies have shown that the presence of chronic kidney disease is an independent risk factor for sudden cardiac death even in the absence of CAD, CHF, and structural heart disease [7]. Chronic renal insufficiency leads to cardiac fibrosis and coronary artery calcification [8-14]. Cardiac structural remodeling in addition to renal disease associated with electrical abnormalities predispose patients to cardiac arrhythmias increasing the risk of sudden cardiac arrest [15]. Renal insufficiency is not only predictive of sudden cardiac death but the presence of acute kidney injury (AKI) in the post-cardiac arrest period has been associated with higher mortality [16]. Cancer patients generally have a higher incidence of AKI as many chemotherapeutic agents are nephrotoxic and these patients are also predisposed to pre-renal azotemia secondary to vomiting and diarrhea induced dehydration [17]. Samuels et al. found that even a small rise in creatinine has been associated with higher morbidity and mortality in critically ill cancer patients [18]. Hepatic dysfunction has also been associated with both systolic and diastolic dysfunction and electrophysiological abnormalities of the heart [19]. We saw that cancer patients who experienced IHCA had higher alanine aminotransferase, alkaline phosphatase, and INR on admission. These parameters have been included in several scoring systems that predict cardiac arrest in general floor patients [5]. Renal and hepatic function tests are available for most patients on admission and these derangements should be given consideration when triaging patients.

Our study also found that patients who did not experience cardiac arrest were more often found to be in normal sinus rhythm based on a 12-lead EKG on admission. Currently, there are no risk stratification calculators based on EKG findings even for the general population; however, high resting heart rate, non-sustained ventricular tachycardia, and heart rate turbulence may be helpful to identify patients at high risk for cardiac arrest when used in combination with other factors [20].

Sepsis was seen more commonly in patients with active cancer. The pathophysiological abnormalities associated with sepsis-like hypovolemia, hypoxemia, and acidosis not only confer a higher risk of cardiac arrest but also are poor predictors of survival after cardiac arrest [21]. Sepsis is known to have higher mortality in patients with cancer than those without cancer [22].

The type of cancer, hematological or solid organ, and the presence of metastatic disease were not predictive of IHCA in our study. Instead, we found that a significantly higher number of patients with cancer who suffered IHCA were not on any treatment for cancer. Most of these patients were newly diagnosed with cancer or had a recently detected recurrence and were scheduled to start treatment. In a study by Bleicher et al., it was found that in patients with breast cancer, a greater time to surgery was associated with lower overall and disease-specific survival [23]. Time from diagnosis to treatment is a vulnerable period and all efforts should be made to shorten it.

CAD is the most common cause of sudden cardiac death [24]. Interestingly, there was no statistically significant difference in the cardiovascular risk factors between the cancer patients who had cardiac arrest compared to patients who did not. Although CAD can cause cardiac arrest through mechanisms other than a myocardial infarction, none of our patients had cardiac arrest as a direct consequence of myocardial infarction [24-25]. Cardiovascular risk factors may have little predictive value for IHCA in cancer patients.

We acknowledge the limitations of our study. Our study was retrospective in nature so there is a possibility of inaccurate reporting of events. Moreoever, it was a single center study with a relatively small sample size. Although multiple studies have looked at the predictors of outcomes after IHCA, this was a first attempt to identify the predictors of having an IHCA in cancer patients. To validate the results of our study, we propose a future multicenter prospective trial where patients can be stratified into high risk versus low-risk category based on the factors identified by our study and then followed to compare the incidence of IHCA.

## Conclusions

Hepatic and renal impairment on admission and the presence of sepsis are predictive of IHCA in patients with active cancer. On the other hand, type of cancer and the presence of metastatic disease did not contribute to the risk of IHCA. Similarly, the presence of cardiovascular risk factors was not associated with higher incidence of IHCA in cancer patients. Patients getting treatment with chemotherapy, immunotherapy and/or radiation therapy within six months preceding the admission were also found to have lower incidence of IHCA. 
